# Interaction effect of Nd:YAG laser and universal adhesive system for dentin sealing 

**DOI:** 10.4317/jced.57306

**Published:** 2020-12-01

**Authors:** Karla-Janilee-de Souza Penha, Carlos-Rocha-Gomes Torres, Rudys-Rodolfo-De Jesus Tavarez, Leily-Macedo Firoozmand

**Affiliations:** 1 DDS, MSc, PhD student, Department of Dentistry, Federal University of Maranhão (UFMA), São Luís, Maranhão, Brazil; 2 DDS, PhD, Professor, Department of Restorative Dentistry, Institute of Science and Technology of São José dos Campos (UNESP), São José dos Campos, SP, Brazil; 3 DDS, MSc, PhD, Professor, Department of Post-Graduation Ceuma University (UNICEUMA), São Luis Maranhão, Brazil; 4 DDS, MSc, PhD, Professor, Department of Dentistry, Federal University of Maranhão (UFMA), São Luís, Maranhão, Brazil

## Abstract

**Background:**

To evaluate the influence of the association of the universal adhesive system to different energy densities of the Nd:YAG laser on the hydraulic conductance of dentin.

**Material and Methods:**

Fifty bovine dentin discs were made. The samples were stratified into four groups (n = 10) according to the treatment performed; SBU- Adper Single Bond Universal (3M ESPE), SBU_60 - SBU associated with the Nd:YAG laser (60mJ, 10Hz, 0.6W - 47.77 J / cm²), SBU_80 - SBU associated with the Nd:YAG laser (80mJ, 10Hz, 0.8 W - 63.69 J / cm2) and C – dentin without treatment (smear layer). Hydraulic conductance measurements were taken 24 hours after the treatments (HC1) and after erosive challenge (HC2). Scanning electron microscopy (SEM) and energy dispersive X-ray spectrometry (EDX) helped to visualize the dentin after the different treatments. Scheffe and Games-Howell statistical tests were used to analyze hydraulic conductance (α = 0.05).

**Results:**

The treated dentin (SBU, SBU_60, and SBU_80) reduced HC1 when compared to dentin with smear layer (C) (*p*< 0.001). The erosive challenge has increased HC2 in SBU_60 and C (*p*< 0.001), and did not promote a significant difference in SBU_80 and SBU. The SEM / EDX analyzes showed an irregular and semi-permeable barrier on the surfaces of the treated dentin.

**Conclusions:**

The association of universal adhesive with Nd:YAG can be an effective alternative for the occlusion of dentinal tubules, whereas higher energy per pulse Nd:YAG (SBU_80) can increase the resistance to permeability when exposed to the erosive challenge.

** Key words:**Dentin, permeability of dentin, Nd:YAG Laser, adhesives.

## Introduction

The increased consumption of erosive diets, dental wear, and occlusal dysfunctions are important factors that can lead to greater exposure of the dentinal tubules (1) . One of the major consequences of dentinal exposure is the prevalence of dentinal hypersensitivity in the adult population (2). Dentin hypersensitivity involves the transmission mechanism of the nervous stimulus through permeability and the movement of fluids in open and exposed dentinal tubules (3). In addition, the opening of the dentinal tubules allows the passage of ions, molecules, fluids, and bacteria from the external environment to the dental pulp.

Exposure of dentinal tubules can occur as a result of pathological conditions, such as; loss of dental enamel with the development of a non-carious cervical lesion, root exposure due to periodontal disease (4), or therapeutic conditions such as cavity preparations performed to receive direct or indirect restorations (5). The obliteration of dentinal tubules has been identified as a potential method for pulp protection (3) and the reduction of dentin hypersensitivity (6).

The interaction between dentin and resin components of adhesive systems form the hybrid layer (7), thus, a significant sealing of dentinal tubules can be observed (8). The universal adhesive systems have been gaining wide acceptance due to their ability to adhere to different surfaces, optimizing direct and indirect clinical treatments. However, these materials undergo greater hydrolytic degradation comparing to 2-step adhesive systems (5,9). In addition, the hybrid layer undergoes enzymatic and proteolytic degradation processes over time (10). The characteristics of universal systems, combined with abrasion and erosion wear can decrease the longevity of tubule sealing by dentin adhesives (1).

The high-power laser, Nd:YAG (Neodymium: Yttrium-Aluminum-Granada) at wavelength: 1064nm, is also capable of obstructing the opening of dentinal tubules (6,11). However, the mechanism of action is unlike the adhesive, as the Nd:YAG acts promoting the “melting” of the dentin surface. This technique secondarily promotes an analgesic effect due to the blockage of fluids from the external environment to the pulp by obliterating the dentinal tubules (12).

Thus, it is proposed to develop a more resistant substrate with the help of fusion of the adhesive to dentin (13). The high power Nd:YAG laser assists in dentin recrystallization and fusion due to the increase in surface temperature, which can optimize the bonding of the universal adhesive (13). Another relevant factor is the help of the penetration of the adhesive in the tubules and the evaporation of solvents and residual water, by increasing the temperature promoted by the laser (14,15), favoring a more sTable tubular seal (16). However, the irradiation parameter used influences the degree of alteration of the dentin substrate (17,18). To perform this technique in order to form a more resistant substrate, it is necessary to evaluate the influence of the association of the universal adhesive with safe laser irradiation parameters to the pulp.

Due to the lack of studies evaluating the behavior of universal adhesives associated with the Nd:YAG laser, the objective of this study is to analyze *in vitro*, the influence of the association of the universal adhesive to the Nd:YAG laser (60 and 80mJ) on the dentin permeability through hydraulic conductance. The null hypotheses tested were: 1) there is no difference in the permeability of dentin treated with Nd: YAG (60 and 80mJ) associated with the universal adhesive system and, 2) the erosive challenge does not promote changes in the dentin permeability of the treated groups.

## Material and Methods

-Sample preparation

Forty freshly extracted bovine incisors from animals aged 2 to 4 years were selected, cleaned, and stored in distilled water for less than 6 months. The teeth were sectioned, below the amelocemental junction, with the aid of a diamond disc (Dremel, Racine, Madison, Wisconsin, USA), adapted to a high-speed lathe (Nevoni, São Paulo, SP, BR), and the roots were despised.

The dental crowns were positioned on a metallic base for the circular cut (Micro Mill, Washington, USA) of the samples. A 6 mm drill was used to delimit the sample diameter, and 1 mm thickness was obtained with the aid of sequential sandpapers P600, P800, P1200 (Fepa P, Extec, Enfield, CT, USA) coupled to the polisher ( DP-10, Panambra, São Paulo, SP, BR) under constant refrigeration (19).

-Measurement of dentin permeability

After preparing the dentin, the smear layer was removed using 0.3% citric acid for 30 sec (20), to reproduce the total opening of the dentinal tubules.

The reading of the dentin permeability in each sample was performed with the aid of the permeability device TDH 03 (ODEME - Equipamentos Médicos e Odontológicas Ltda, Joaçaba, SC, BR). Dentin permeability was determined by hydraulic conductance (Lp), dentinal fluid flow, determined by the formula: Lp = Q / (SA.P), where Lp = hydraulic conductance expressed in µl.cm-2.min-1. cmH2O-1, Q = infiltration rate in μL.min-1, SA = surface area exposed to infiltration in cm², *P* = hydrostatic pressure in dentin in cmH2O (19).

To establish differences in dentin permeability, hydraulic conductance was measured in three moments: after removal of the smear layer - initial hydraulic conductance (HC-0) (PI), after treatments (application of adhesive systems + laser) (HC -1) and after erosive challenge (HC-2).

**Figure 1:**
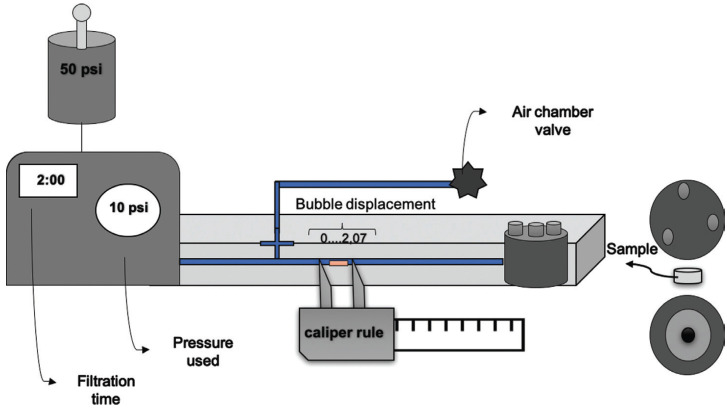
Schematic drawing of the permeability device.

In the permeability apparatus, for measuring hydraulic conductance, disk-shaped samples were placed between two rings that allowed standardizing the dentin area available for filtration of deionized water (0.03801 cm²) and adequate sealing. The dentin surface corresponding to the pulp chamber was placed in contact with the fluid (deionized water) under the pressure of 703 cmH2O and the external side facing upwards, according to the dental structure. The filtration of the fluid through the dentin was followed by 2 min by linear displacement of an air bubble inserted in the glass capillary using a digital caliper (19). The glass capillary (internal volume of 75 µl and length of 101 mm) joins the water reservoir to the perfusion chamber, allowing the calculation of the fluid perfusion rate through the dentin (Fig. 1). Three consecutive measurements of the linear displacement of the bubble were recorded for each.

-Dentin treatment (experimental groups)

From the results of the initial hydraulic conductance reading (HC-0), the samples were stratified, divided into 5 groups (n = 10), so they presented equivalent HC-0 means. Dentin surfaces were treated according to the following techniques (n = 10); SBU- Adper Single Bond Universal self-etching adhesive system (3M ESPE, Seefeld, Germany), SBU_60- Self-etching adhesive system associated with Nd:YAG laser irradiation at 60 mJ / 10 Hz for 60 seconds, SBU_80- Self-etching adhesive associated with Nd:YAG laser irradiation at 80 mJ/ 10 Hz for 60 seconds and C- control; dentin with smear layer (without treatment).

The adhesive system Single Bond Universal (3M ESPE, Seefeld, Germany: Lote1306600524) was applied in 2 layers on dentin, actively, for 20 seconds according to the manufacturer’s instructions, in groups SBU, SBU_60, and SBU_80.

For groups treated with the Nd: YAG laser (Neodymium: Yttrium-Aluminum-Garnet), the Pulse Master 600 IQ device (American Dental Technologies, Austin, Texas, USA) was used, with λ 1064nm and the optical fiber of 400 μm. The irradiation was performed on the adhesive applied to the dentin before its light curing. A surface scan was performed, in four 45° spaced directions (vertical, horizontal and two transversal), for 40 seconds with the optical fiber positioned perpendicular to the dentin surface, promoting homogeneous irradiation.

The parameters used in the irradiated groups were; 60 mJ of energy per pulse, 10 Hz (or 10 frequency) and 0.6 W of power - energy density 47.77 J / cm² for (SBU_60), and 80 mJ of energy per pulse, 10 Hz (or 10 frequency) and 0.8W of power - energy density (63.69 J / cm²) for (SBU_80).

The adhesives were light-cured with an LED-Elipar Free Light 2 light curing device (3M ESPE St. Paul, Minnesota, USA) with an irradiance of 700 mW / cm², for 20 seconds.

After the treatments, the samples were stored in deionized water at 37 ± 1 ° C for 24 h.

-Erosive Challenge 

In order to evaluate the behavior of this new substrate against erosion challenges, four daily erosion cycles were carried out for five days. After treatment on the buccal surface of the dentin discs (21), these surfaces were subjected to erosive cycles that consisted of immersing the samples in 0.3% citric acid solution at pH 2.3 for 2 minutes, followed by washing in ultrapure water and immersion in artificial saliva (pH = 7) for 1 hour (20). Before starting a new cycle, the samples were washed with ultrapure water and new solutions of citric acid and artificial saliva were used. The sequence of 4 erosion challenge cycles and 20 hours of immersion in artificial saliva was repeated for 5 consecutive days.

At the end of the erosive cycle, the samples were stored in identified eppendorfs containing ultrapure water in an oven at 37 ± 1°C.

-Calculation of permeability in percentage after treatment and erosion challenge

Dentin permeability after treatments and erosion challenge was calculated as a percentage (%) with reference to the maximum permeability of each sample. The following formula was applied: P% = (Lp / LpMax) x 100 (21).

The values represent P% = percentage of permeability, conductance value after treatment (Lptreat), or after erosive challenge (Lpec) and Lpmax = maximum value of hydraulic conductance with open tubules. Thus, the percentages of hydraulic conductance of% PPTrat (percentage of hydraulic conductance after treatment) and% PPec (percentage of hydraulic conductance after erosive challenge) were calculated and obtained.

To reduce the inherent variability of dentinal tissue (size and quantity of tubules) (22), each disc was its own control (21,22), and a control group (dentin with smear layer) was delimited to allow for comparison of this substrate to the treatments carried out.

-Scanning Electron Microscopy / Energy Dispersive X-ray Spectroscopy (SEM / EDX)

Micrographs (3000x) were taken after applying the adhesive systems and after the erosive challenge, to observe the behavior of different types of treatment on dentin. For this, the samples were dried in serial sequences of alcohol and placed in a desiccator for 24 h. Subsequently, the samples were placed on aluminum stubs with the aid of a carbon conductive tape and taken to the metallization device (SC7620 Sputter Coater, Emitech).

The Scanning Electron Microscope (SEM) (Inspect S50 -FEI, Czech Republic) using 25 KV and EDS (JMS 5310-Jeol Brasil Instruments Científicos Ltda. - SP, BR) was used to evaluate the quality of dentin after treatments and erosive challenge. The analysis of the samples was performed capturing the images through software coupled to SEM (Inspect 550, Fei).

-Statistical analysis

The mean values (standard deviations) and median of% PPTrat and% PPec were calculated. The normal distribution and the assumption of equality of the variances were verified using the Levene test (*p* = 0.000). The data were analyzed using two-way ANOVA followed by the Scheffe or Games-Howell post-Hoc test (*p* = 0.05).

The statistical program used was SPSS 25.0 (IBM, Armonk, NY, USA). The level of significance adopted was 5%.

## Results

-Dentin Permeability Analysis

**Figure 2:**
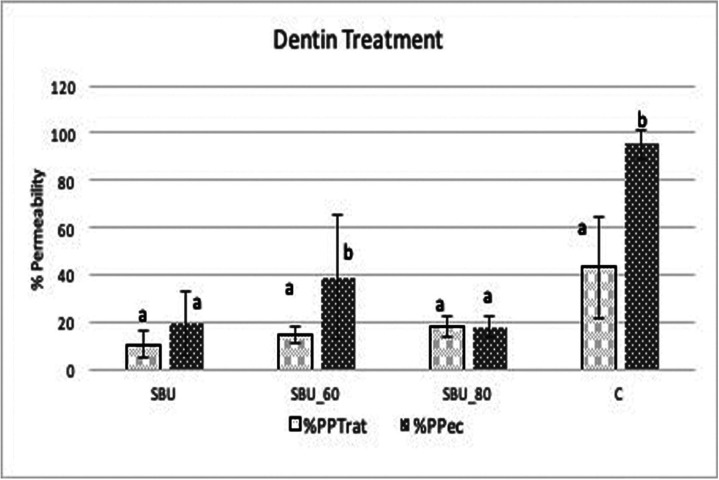
Mean and standard deviation of the % permeability of the treated dentins (% PPTrat) and after the erosive challenge (% PPec).

The mean values of the percentage change in permeability P% and standard deviation after treatments (% PPTrat) and erosive challenge (% PPec) are shown in Figure 2.

Treated dentin (SBU, SBU_60, and SBU_80) had a significant reduction in permeability when compared to dentin with smear layer (C) (*p* = 0.000). The use of universal adhesive (SBU) reduced dentin permeability in the same way as when associated with Nd:YAG. However, after the erosive challenge, SBU tended not to alter the permeability (*p* = 0.057). The erosive challenge increased permeability of the SBU-60 (*p* = 0.021) and C (*p* = 0.000) groups, and did not significantly change the dentin treated with SBU_80 (*p* = 0.0488) and SBU (*p* = 0.057).

Although the irradiation parameters used (60mJ and 80mJ) did not show a significant difference between them (*p* = 0.406), only Nd: YAG (80mJ) was able to maintain the permeability values after the erosive challenge (*p* = 0.488).

-Scanning Electron Microscopy / Energy Dispersive X-ray Spectroscopy (SEM / EDX) Analysis

**Figure 3:**
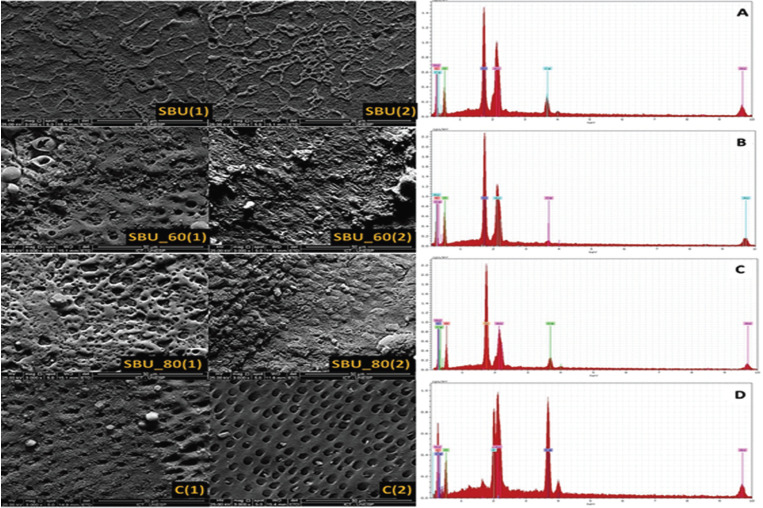
SEM micrographs of the treated dentin surfaces (3000x). (1) after 24h of treatment and (2) after the erosive challenge. A (SBU), B (SBU_60), C (SBU_80), D (Control).

Photomicrographs illustrate the dentinal tubules after treatment and erosion challenge (Fig. 3). A uniform layer with less porosity was found in SBU when compared to the adhesive associated with the Nd:YAG laser (SBU_60 and SBU_80). SBU_80 presented a layer with apparent interaction under the dentin surface, this characteristic was more evident after the acid challenge. The interaction, with partial obliteration of the tubules, was also observed in SBU_60.

In group C, after the erosive challenge, removal of the smear layer and complete exposure of the dentinal tubules were observed (Fig. 3).

EDX allowed observing the presence of silicon (Si) in the groups treated with adhesive, demonstrating its presence even after irradiation with Nd: YAG laser. Larger peaks of Calcium (Ca) and phosphorus (P) are found in untreated dentin (C). Less Ca was found in lower energy densities (SBU_60) when compared to higher energy density (SBU_80).

## Discussion

The dental substrate is exposed to frequent acid-mechanical challenges in the oral environment, so the formation of a more resistant substrate would be a viable strategy for the prevention and treatment of exposed and hypersensitive dentin. The association of the Nd:YAG laser with the universal adhesive system, were able to obliterate the dentinal tubules when compared with the untreated dentin, so the first hypothesis was rejected. The erosive challenge increased the permeability of groups SBU_60 and C, rejecting the second hypothesis of the study.

The Nd:YAG pulse energies of 60 mJ and 80 mJ was employed in this study because it is reported in the literature that these energies promote respectively, a temperature increase of 3.90 ° C and 4.80°C (21). These temperature increases inside the pulp chamber are according to increase tolerated by the pulp (below 5.5°C) (23). Therefore, high energy density values would impair the pulp’s vitality, in addition to promoting dentin cracks, as verified in the pilot study previously performed. Pulse energies greater than 80 mJ formed cracks and fractures, making the dentin more porous and permeable and those less than 60 mJ did not cause changes in the dentin, consolidating the choice of parameters used in this study (21).

Clinical studies indicate that the application of the Nd: YAG laser, and the use of the adhesive system on dentin, present an immediate reduction in dentinal hypersensitivity (6), corroborating the reduction in permeability observed in our study. Clinically, the Nd: YAG laser was more effective for hypersensitivity when compared to adhesive systems and low power lasers (6). Higher values of bond strength after erosive challenge were reported for dentin irradiated with Nd: YAG (24) raising the hypothesis that the laser could act as a barrier resistant to dissolution by the acid challenge.

The universal adhesive system is widely accepted for its ease of handling and a large range of clinical use. The SBU did not completely seal the dentin, allowing the passage of dentinal fluid, as verified in the literature (5). The SBU permeability values reflect the influence of the 10-MDP monomer that promotes a chemical nano-interaction with the hydroxyapatite, allowing a more sTable tubular obliteration (25,26). As verified in SEM, a resin barrier forms on the dentin, however, the performance of these universal adhesives is material dependent and often inferior to the 2-step adhesives (27), mainly over time (9). The results of this study revealed that after the erosive challenge, there was a tendency to increase permeability. It is suggested that longer periods of acid challenge and/or conditions that allow hydrolytic, enzymatic, and proteolytic degradation could increase the permeability of one-step adhesive systems.

Dentin impregnated with the unpolymerized adhesive associated with the Nd: YAG laser can increase the adhesive strength and recrystallization of dentin, as suggested by Gonçalves *et al.* (13), contributing to the formation of a more uniform layer (14). This association has demonstrated better values of adhesive resistance in the dentin (14,17). Also, hot air jets can increase solvent evaporation and improve material performance in the short and long term (28), and this peculiarity is achieved with the use of the Nd:YAG laser (15).

The results of the present study demonstrated that despite the association of Nd: YAG (60 and 80 mJ) reducing the permeability when compared to dentin with smear layer, SBU_60 and SBU_80 did not present statistical differences between them. The greater energy density (SBU_80) guaranteed greater permeability stability after the erosion challenge. Nd:YAG laser does not alter the calcium concentrations of dentin (29), so chemical interaction of the universal adhesive with dentin can be established and the formation of resinous tags is not compromised (14). Besides, Nd:YAG laser when used on non-polymerized adhesive, helps solvent evaporation (15), changes the organic content, collagen (amide I, II and III) and reduces water (30). It is suggested that these factors may decrease the chances of hydrolytic and proteolytic degradation of the hybrid layer. Previous studies indicate that the association of Nd:YAG with adhesives (14,15), may form a new recrystallized hydroxyapatite compound associated with the melted adhesive (13).

It was observed that among the safe energy densities for the pulp (21), SBU_80 was able to decrease dentin permeability and maintain it even after the erosive challenge. The higher amount of (Ca) verified in EDX, in the dentin surface layer treated with higher energy density (SBU_80) when compared to SBU_60, may suggest a greater integration of the adhesive with the dentin, resulting in stability of the permeability values even after the erosive challenge.

## Conclusions

According to the limitations of this study and the results obtained, it is concluded that the universal adhesive system, and the association of Nd:YAG laser irradiation (60mJ and 80 mJ) associated with the universal adhesive, reduce dentin permeability when compared to untreated dentin. Also, higher energy density (SBU_80), can result in greater resistance to permeability of dentin exposed to erosive challenge.
